# Can We Efficiently Target HDAC in Cancer?

**DOI:** 10.3390/cancers14164058

**Published:** 2022-08-22

**Authors:** Tobias Kiesslich, Daniel Neureiter

**Affiliations:** 1 Center for Physiology, Pathophysiology and Biophysics-Salzburg and Nuremberg, Institute for Physiology and Pathophysiology-Salzburg, Paracelsus Medical University, Strubergasse 22, 5020 Salzburg, Austria; 2 Department of Internal Medicine I, University Clinics Salzburg, Paracelsus Medical University, Müllner Hauptstrasse 48, 5020 Salzburg, Austria; 3 Institute of Pathology, University Clinics Salzburg, Paracelsus Medical University, Müllner Hauptstrasse 48, 5020 Salzburg, Austria; 4 Cancer Cluster Salzburg, 5020 Salzburg, Austria

According to the hallmarks of cancer, typical processes of human cancer initiation, progression, and metastasis are essentially influenced by pathologic epigenetic deregulations via DNA methylation and/or histone modification [1,2], leading to the recently introduced term of epigenetic programming of hallmark cancer phenotype [3].

In particular, modifications of histones—such as histone de-/acetylation—represent a central epigenetic regulatory mechanism which leads to relevant alterations of chromatin structure due to dysregulated noncovalent interactions within and between nucleosomes, and play a significant role in human carcinogenesis [4,5]. Histone deacetylation processes are regulated by a group of related enzymes called histone deacetylases (HDACs), categorized in 4 classes with 18 reported HDACs [6]. Heterogeneous up- and downregulation of HDACs has been reported in several human cancer types, making these regulative enzymes a very interesting new potential therapeutic target [7]. HDACs also regulate the posttranslational acetylation status of a variety of other nonhistone substrates including key tumor oncogenic and suppressive genes, as wells as associated proteins involved in mRNA stability, protein localization and degradation, and protein–protein and protein–DNA interactions [8,9].

Regarding our knowledge of the role of HDACs in human carcinogenesis, more than thirty years of developing HDAC inhibitors have passed since the first report of a potent zinc-dependent histone deacetylase [10]. To date, most of the developed synthetic histone deacetylase inhibitors (HDACis) are not HDAC class- or HDAC class member-specific [6]. Therefore, the development of new, highly selective histone deacetylase inhibitors (such as the HDAC6-specific inhibitor Rocilinostat or the HDAC8-specific inhibitor Trapoxin) is a challenging and very promising scientific field. Additionally, treatment strategies combining HDACis with approved anticancer drugs could essentially improve the therapeutic success rate through inhibition of the tumor resistance mechanism or re-induction of primarily epigenetically silenced and therefore possibly druggable proteins in future [9,11,12]. The background of HDACs and their involvement in cancer is illustrated in Figure 1.

In what follows, we try to address some specific points and issues of the papers (three original and two review articles) published in this Special Issue to highlight the great potency to target deregulated HDACs in human cancer:

(i) *Mayr* et al. investigated the HDAC expression in biliary tract cancer (BTC) in relation to effects of HDAC inhibitors in-vitro (using established BTC cell lines) and to clinical endpoints in-situ (using BTC specimens). They could demonstrate that: (i) HDAC inhibition show a significant cytotoxic effect alone; and that (ii) especially the HDAC class I (HDACs 1, 2) inhibitor romidepsin could significantly augmented the cytotoxic effect of the standard chemotherapeutic cisplatin on the BTC cell lines. Furthermore, HDAC2 expression in human BTC specimen could be used as the diagnostic and prognostic biomarker. From this study, the conclusion could be that HDAC inhibition might be a promising combinatory drug approach in BTC as a cancer entity with dismal prognosis and that the tissue expression levels of HDACs seems to be an interesting biomarker in BTC [13].
(ii) *Maria Hernandez-Valladares* et al. studied the in vivo proteomic/phosphoproteomic effects of all-trans retinoic acid (ATRA) and valproic acid (VP) on primary acute myeloid leukemia cells derived from patients before and during disease-stabilizing treatment. Prior to epigenetic therapy, the proteome and phosphoproteome profiling of the AML responders/non-responders cases revealed significant difference in processes of neutrophil degranulation/differentiation, M phase regulation and the interconversion of nucleotide di- and triphosphates (i.e., DNA synthesis and binding), as well as RNA metabolism and apoptosis. Treatment of AML cell lines with the combination of ATRA and VP leads to proteomic and phosphoproteomic modulation of DNA strand elongation, RNA processing, actin/cytoskeleton and cholesterol metabolism as well as GTPase/intracellular signaling in relation to results from AML responders and non-responders. Therefore, the authors suggested that proteomic and phosphoproteomic profiling can be applied as a predictive “tool” for identification of responders to ATRA/VP-based treatment [14].
(iii) *Sanne Venneker* et al. investigated the changes of the methylome from IDH-mutant enchondroma towards high-grade chondrosarcoma in situ and performed an epigenetics and combinatory compound screening in chondrosarcoma cell lines in vitro, too. They could show that: (i) methylation is increased with the grading of IDH-mutant chondroid-derived neoplasm; and (ii) that chondrosarcoma cell lines were sensitive to HDAC inhibition in 2D and 3D in vitro models independent to their IDH mutation status. Detailed analysis with drug screenings revealed specific pro-apoptotic effects of class 1 HDAC inhibition on chondrosarcoma cells leading to synergistic effects in the combination with Bcl-2 family member inhibitors. Therefore, the authors concluded that combination therapies of specific HDAC inhibitors and small molecule inhibitors could be a promising strategy to improve therapeutic efficacy in chondrosarcoma [15].
(iv) *Robert Jenke* et al. provided an up-to-date, comprehensive, and very structured overview of recent status and ongoing developments of HDAC inhibitors in the field of anticancer therapy. They summarized the molecular mechanisms of HDACi-linked anticancer effects to “classically” induce apoptosis and autophagy as well as senescence, whereby new insights on DNA damage, hormone signaling, and immune regulation by HDACi are presented. Regarding therapeutic aspects, the authors of this review display how combinatory approaches of HDACi with phosphoinositide 3-kinase-EGFR inhibitors and hormone or immunotherapy could improve therapeutic efficacy. Finally, very interesting developments regarding new bifunctional inhibitors as well as novel approaches for HDAC degradation via PROteolysis-TArgeting Chimeras (PROTACs) are discussed in detail. Based on the pre-clinical and clinical results of bifunctional HDAC inhibitors, the authors postulate that the upcoming concept of polypharmacy with HDACs is very promising for the field of cancer therapy [16].
(v) *Hélène Losson* et al. focus in their review on the possible role of HDAC6 as a new and clinically relevant target in chronic myeloid leukemia (CML). First of all, the authors could definitively demonstrate the specific involvement of HDAC6 in human oncogenesis of human tumors via different mode of actions. Looking in detail at CML: (i) nuclear shuttling of HDAC6 with p53-HDAC6 interactions; (ii) the HDAC6 related acetylation status of HSP90 for degradation of BCR-ABL; and (iii) the overexpression of HDAC6 in CML stem cells make especially HDAC6 an interesting target in CML [17].

Regarding the question of this editorial, “Can we efficiently target HDAC in cancer?”, the answer after summarizing the main results and conclusions of the published manuscript in this special issue is a clear “yes”. Yes, we can target HDAC in human cancer in a specific matter in order to adequately induce tumor-related cytotoxicity alone or possibly better in a combinatory strategy [13,14,15,16,17]. However, which issues should be addressed in the future? Most findings are based on pre-clinical experimental designs with human cancer cells in-vitro and in-vivo. The transfer from the bench to the bedside is ongoing and challenging. A recent query with the terms “HDAC” and “malignancy” in the clinical trial data database of the NIH (https://clinicaltrials.gov, accessed on 12 August 2022) returned 188 hits overall (including 94 completed and 15 recruiting clinical trials) indicating the fact that HDAC inhibitors have now arrived in clinical research. However, the pace of implementation for definitive clinical practice is still moderate. It is worth looking at the currently recruiting trials (see Table 1): HDAC inhibitors are applied for heterogeneous tumor entities ranging from solid tumors to different hematological malignancies: (i) sometimes in orphan or rare diseases; and (ii) mostly in advanced and/or in relapsed/therapy-refractory stages. This could the reason that the therapeutical effects of HDACs in these clinical trials are limited in relation to clinical endpoints and to recommended first, second, or third-line therapy strategies. The results of these clinical trials combining HDACs and immunomodulating substances or immune checkpoint inhibitors will be interesting in relation to the our knowledge of epigenetic regulation of the tumor microenvironment including stroma and immune cells [3]. 

Nevertheless, there is still a definitive need for action here to obtain more insights concerning the efficacy of HDACis in humans regarding polypharmacy and combinatory treatment strategies in relation to timing, sequencing, and intensity [18].

Therefore, the articles in this Special Issue are intended to give some potential mechanistic insights of HDAC inhibitor using in vitro experiment with the aggressive tumor entities of biliary tract cancer, acute myeloid leukemia and chondrosarcoma. Furthermore, additional comprehensive reviews could demonstrate how HDACs are definitively involved in human cancerogenesis and how the new approach of polypharmacy could help to overcome deregulated epigenetics by specifically targeting HDACs. We sincerely hope that the articles in this Special Issue will inspire the cancers audience to enforce our common fight against human cancer now and in the future and to give the cancer suffering patients more hope for potential healing.

## Figures and Tables

**Figure 1:**
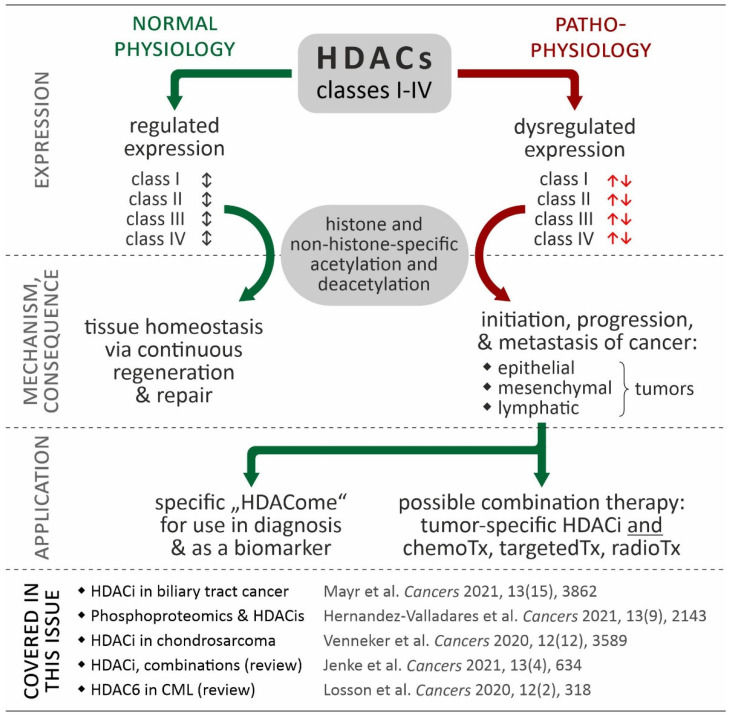
Relevance of HDACs in cancer. While coordinated in normal physiological processes (black double arrows), in many cancers, HDAC isoforms show de-regulated expression contributing to the hallmarks of cancer (red arrows). Based in this data, the “HDACome” on individual cancer types could be used for diagnosis (e.g., biomarker) or be exploited in tumor-specific combination of HDACi and other therapies. The references provided in the figure refer to articles in the special issue “Targeting Histone Deacetylases in Cancer” (Cancers, https://www.mdpi.com/journal/cancers/special_issues/histone_deacetylases, accessed on 12 August 2022) [13,14,15,16,17].

**Table 1:** Recruiting clinical trials dealing with HDAC inhibitors and malignant disease (regarding the terms “HDAC” and “malignancy” in the clinical trial data database of the NIH, see https://clinicaltrials.gov (accessed on 12 August 2022); clinical trial #7 does not apply an HDAC-inhibitor, but include the term HDAC in the list of keywords. Abbreviations: 5-Aza = 5-Azacitidine, A = advanced, DNMTi = DNA methyltransferase inhibitor, E = early, n.a. = not applicable, NSCLC = non-small cell lung cancer, R = relapsed/refractory.

Rank	Tumor Entities	Tumor Stage	HDACi	Target (s)	Combinatory Drug Intervention	Phase	Identifier (NCT-):
**1**	Cervical cancer	A	**Chidamide**	class I, IIb	Toripalimab (PD-1 Inhibitor)	1–2	04651127
**2**	Solid tumors	A	**JBI-802**	HDAC6		1–2	05268666
**3**	Breast and ovarian cancer	A/R	**Belinostat**	pan	Ribociclib (inhibitor of cyclin D1/CDK4 and CDK6)	1	04315233
**4**	Multiple myeloma	E	**Citarinostat**	AC6	Hiltonol (immunostimulant), Lenalidomide (immune modulation), PVX-410 (multi-peptide vaccine)	1	02886065
**5**	Advanced solid tumors	A	**Tinostamustine**	class I		1-2	03345485
**6**	Hematologic malignancies	R				1	02576496
**7**	NSCLC and esophageal carcinomas	A	**Decitabine (a DNMTi),** **Tetrahydrouridine (inhibitor of cytidine deaminase)**	n.a.	Pembrolizumab (PD-1 Inhibitor)	1–2	03233724
**8**	T-cell malignancies	R	**Romidepsin**	class I	Lenalidomide (immune modulation), 5-Aza (DNA methyltransferase inhibitor)	1	04447027
**9**	Follicular lymphoma	R	**Abexinostat**	pan		2	03934567
**10**	Extrapulmonary neuroendocrine carcinoma	A	**Chidamide**	class I, IIb	Etoposide (DNA topoisomerase II-inhibitor), Cisplatin/ Carboplatin (DNA replication interfering)	2	05076786
**11**	Neuroendocrine neoplasm	A			Sintilimab (PD-1 inhibitor)	2	05113355
**12**	T-cell lymphoma	n.a.				2	04512534
**13**	Extranodal NK/T-cell Lymphoma	n.a.				2	04994210
**14**	Multiple myeloma	R	**HG146**			1	03710915
**15**	Adult Ph-like ALL	n.a.	**Chidamide**		Dasatinib (tryosinkinase inhibitor)	2–3	03564470

Abbreviations: 5-Aza = 5-Azacitidine, A = advanced, DNMTi = DNA methyltransferase inhibitor, E = early, n.a. = not applicable, NK = natural killer cell, NSCLC = non-small cell lung cancer, R = relapsed/refractory.
